# Quantitative Systems Pharmacology Modeling Amid the Rise of Agentic AI

**DOI:** 10.1002/psp4.70249

**Published:** 2026-04-09

**Authors:** James Lu, Rajat Desikan

**Affiliations:** ^1^ Bioinformatics Institute (BII), Agency for Science Technology and Research (A*STAR) Singapore Singapore; ^2^ Clinical Pharmacology & Pharmacometrics Systems and Tools Platform Sciences, GSK Stevenage UK

**Keywords:** agentic AI, AI/ML, LLM, QSP

The rapid advancement of artificial intelligence (AI) is creating a paradigm shift in scientific research. In particular, agentic AI systems, with their nascent capabilities of reasoning and computational tool use, are now beginning to take an active role in the process of making scientific discoveries. In the field of quantitative systems pharmacology (QSP), modelers have long grappled with a fundamental challenge: on the one hand, the need to develop computational models which are sufficiently high‐fidelity in recapitulating the complexity of the underlying biological and disease processes; on the other hand, the need for the models to be actionable and delivered in a timely manner in settings such as drug discovery & development. The tension between granularity and utility has long been considered a dichotomy in QSP, requiring a decision to be made regarding the right trade‐off. The rise of agentic AI marks a critical juncture that invites us, as Associate Editors of CPT:PSP, to revisit long‐held assumptions and warrants a reflection on the paths ahead for the field.

The recent paper by Saini and Farnoud [1] published in CPT:PSP is a pioneering example of leveraging agentic AI as a co‐pilot to improve QSP modeling workflows. It signals the early dawn of a new era anticipated by Androulakis et al. [2], whereby large language models (LLMs) are elevated from being computational tools to becoming partners [3] in the area of QSP modeling. Here, we consider how agentic AI can best be integrated to solve the key challenges of the discipline from a broader perspective.

## 1 Brief Retrospective: QSP As Using Mathematics & Computation to Understand Diseases and Treatments

The foundational 2011 NIH White Paper [4] articulated the following ambitious aims for QSP to advance drug development and improve human health: (1) to integrate quantitative, systems‐level approaches with classical pharmacology, replacing the “one‐gene, one‐receptor” paradigm to uncover fundamental network‐centric understanding of drug action; (2) to develop multi‐scale computational models spanning molecular mechanisms to whole‐patient responses, bridging the critical gap between preclinical mechanistic studies and clinical pharmacokinetic/pharmacodynamic outcomes; (3) to provide an integrated systems‐level approach to investigating variability in therapeutic and toxic drug responses at single‐cell, organ, and patient levels through differences in proteome, genome, and environment, and thereby unravel novel pharmacodynamic biomarkers; and (4) to improve clinical trial success rates, particularly Phase II/III efficacy trials, through better target validation, predictive models, systematic failure analysis methodologies, rational design of combination therapies, and network‐level prediction of on‐target and off‐target toxicity.

However, the impact of QSP has been uneven: while substantial progress has been made in clinical development settings (Aim 4) and modest advances achieved in translating preclinical findings to humans (Aim 3) [5], there has been limited success in target identification and prioritization, discovery of novel mechanisms of action through systems analysis, biomarker identification, or more broadly in uncovering new biology and proposing novel therapeutic mechanisms.

The limited progress of QSP toward Aims 1 and 2 can be attributed primarily to two fundamental challenges: technical hurdles and resource requirements. Firstly, true platform drug‐disease QSP models, such as models of the human adaptive immune response to vaccines or the tumor microenvironment across cancer types, are extremely challenging to construct due to the immense complexity of biological networks that remain incompletely understood (i.e., epistemic gaps). Additionally, there are technical difficulties integrating currently available multi‐modal data (e.g., omics, imaging, electronic health records, pharmacogenomics) into QSP models to mechanistically link biological processes across spatial and temporal scales. Related to this are challenges that arise from data heterogeneity, assay variability, sparsity, incomplete information, and fundamental mathematical limitations of ODE‐based models in capturing some of the data and biological processes. The second fundamental challenge is that QSP model development is labor‐intensive and time‐consuming, requiring multidisciplinary teams to manually curate extensive published literature, biological process maps, and databases over months to years. This effort is necessary to construct mechanistically accurate, validated models capable of extrapolating into novel biological regimes, such as for mechanistic hypothesis testing and prediction of novel combination therapies.

While these have been longstanding challenges limiting the broader impact of QSP modeling, recent advances in AI mark the beginning of a new phase in how such models may be constructed and applied.

## 2 Hyperscale Data and Compute Giving Rise to Biomedical AI

In parallel to the advancements in QSP models and their impacts, over the recent years we have witnessed how advancements in AI architectures (such as Transformers) and training algorithms have enabled the ingestion of massive amounts of texts, images and videos into AI models. Through self‐supervised learning, emergent capabilities have arisen in these AI models to allow them to perform complex tasks such as language understanding, coding computer programs, and many others. The capabilities of AI models in the technical and scientific domains have also been dramatically rising, an example being the demonstrated ability to solve highly challenging mathematical problems at competitive levels [6].

The biomedical domain is no exception to this transformation: the rapid growth of the volume of biological data and advances in computational power are driving the emergence of biomedical AI [7]. In particular, technological advancements ranging from omics, medical imaging, electronic health records, wearable sensors, and many others have produced vast amounts of high‐dimensional data that capture the complexities of human diseases in unprecedented detail. In parallel, advancements in accelerated computing such as Graphical Processing Units (GPUs) have led to an exponential increase in the amount of compute available per dollar. Taken together, the resulting biomedical AI is poised to rapidly increase its capability and open new avenues for disease understanding and therapeutic insights that were not previously possible. This emerging opportunity is reflected by an increase in research funding as well as commercial activity in using AI to discover new targets and streamline clinical development from multimodal biomedical data.

## 3 From Seeing to Reading, Reasoning and Acting: The Emergence of Agentic AI

With the increased importance of machine learning (ML) in scientific applications, the pharmacometrics and systems pharmacology (PSP) community has also developed and applied these algorithms to various modeling tasks [3, 8]. Much of these efforts have involved replacing well‐established linear and other empirical parametric models (such as the *E*max function) with those represented by trees and neural networks [9] which *learn* from the available data. As such, these ML models can be used to identify covariate relationships that are nonlinear and thereby potentially provide additional insights as well as being possibly less biased than existing empirical models [10].

The PSP community relies significantly on visualization techniques to understand data and assess the quality of model fits to data. One of the key opportunities for leveraging AI is its capacity to “see” patterns in high‐dimensional data beyond what can be readily visualized by the human eye. There have been encouraging results demonstrating that deep learning models applied to biomedical imaging data can pick up salient features that are predictive of patients' physiological and/or disease status which may be missed by even well‐trained human experts. While published studies have demonstrated the benefits of ML for predictive tasks, there has also been considerable caution in the adoption of such algorithms within the PSP community, driven in part by factors such as departure from traditional modeling assumptions, insufficient prior experience and trust with these algorithms, particularly the interpretability challenge of these complex models.

While the PSP community has continued to grapple with the adoption and application of AI/ML algorithms, the emergence of LLMs has rather suddenly enabled AI systems to read scientific articles and reason about scientific concepts in ways arguably analogous to human modelers. Additionally, programming codes and mathematical equations have become part of the analytical language through which the AI systems are able to express their outputs. Via its ability to use computational tools and analyze the results, AI is able to turn reasoning into action and deliver models that are expressed not in terms of trees and neural networks, but in a language familiar to the modeling community: systems of differential equations.

## 4 Agentic AI for QSP

The QSP‐Copilot paper [1] by Saini and Farnoud represents a landmark development at the intersection of QSP and agentic AI. The platform employs a multi‐agent system architecture where autonomous agents possess decision‐making capabilities, tool access (code execution, database queries, API calls), and both short‐term memory for tracking reasoning chains and long‐term memory for project‐level context. Combined with retrieval‐augmented generation (RAG) from curated databases (e.g., scientific literature—PubMed, Google Scholar; biological pathways—Reactome, STRING; protein and drug databases—UniProt, DrugBank, etc.) and multiple large language models (GPT‐4o, Claude Sonnet, TxGemma), QSP‐Copilot orchestrates a near‐autonomous QSP workflow from literature extraction through model structuring to equation generation while maintaining human oversight through mandatory validation checkpoints. In applications to rare disease modeling (blood coagulation and Gaucher disease), the platform achieved high precision in biological mechanism extraction and reduced development time by ~40% for knowledge synthesis from literature into a QSP model diagram.

However, while QSP‐Copilot currently generates base models with placeholder parameters requiring subsequent calibration, the entire QSP end‐to‐end modeling process is not feasible yet. New features such as automated parameter estimation, virtual population generation and calibration, designing and running in silico trials, analysis and visualization are among the capabilities necessary for an end‐to‐end QSP workflow. Further, benchmarking with state‐of‐the‐art QSP models across therapeutic areas and improvements for robustness, reproducibility, and reducing hallucinations are also necessary before broad deployment of these tools within drug discovery and development. However, agentic AI is a rapidly evolving field, and tools such as QSP‐Copilot should not be treated as static snapshots but rather as dynamic frameworks whose core features will quickly grow in number and capability, thus enabling next‐generation QSP models.

## 5 Redrawing the Boundaries of What QSP Could Do

### 5.1 Efficiency and Scalability

Recently, Swanson et al. [11] demonstrated a fundamental advance in AI‐augmented scientific discovery through their “Virtual Lab” framework, where a team of specialized AI agents orchestrated by a Principal Investigator agent performed sophisticated, open‐ended interdisciplinary research with minimal human oversight. Unlike prior applications of LLMs that primarily answer specific scientific questions, the Virtual Lab autonomously navigated a complete research workflow: namely, assembling an interdisciplinary team, selecting and implementing computational tools, designing a novel analytical pipeline, and ultimately producing experimentally validated real‐world discoveries. The human researcher contributed only 1.3% of the total input by word count, providing strategic guidance while the AI agents handled the technical execution, completing the computational design work in days rather than the weeks typically required. This represents a paradigm shift for AI's role in science, from a tool that answers questions to a collaborative system capable of executing complex research projects from conception through experimental validation.

The current version of QSP‐Copilot as described in [1] addresses primarily the knowledge synthesis phase of the QSP modeling lifecycle. While QSP‐Copilot currently generates models with placeholder parameters and base ODE structures, future agents could autonomously estimate parameters from literature and databases, incorporate cellular dynamics when appropriate, and propose alternative model structures for critical evaluation. Beyond literature synthesis demonstrated in QSP‐Copilot, future AI‐QSP systems may ingest multimodal experimental data—transcriptomics, proteomics, imaging, clinical measurements—directly analyze these datasets and generate novel mechanistic hypotheses for computational testing. This capability would enable QSP models to serve as dynamic computational laboratories that not only incorporate emerging knowledge but actively participate in the hypothesis‐generation cycle, potentially guiding experimental design and resource allocation.

A fundamental limitation of frameworks such as QSP‐Copilot stems from its reliance on LLMs as the underlying reasoning engine, whose probabilistic and non‐deterministic nature means that identical prompts can yield different model structures, parameterisations, or code across runs, undermining the reproducibility expected in regulatory‐standard modeling. Hallucinations pose a particularly insidious risk in this domain, as an LLM may generate plausible looking but pharmacologically incorrect ODEs, fabricate parameter values, or cite non‐existent literature to justify model assumptions, and these errors may escape detection if not checked by deep domain expertise. Perhaps most critically, the opaque and stochastic decision‐making process frustrates the establishment of a rigorous model audit trail. Unlike a human modeler whose rationale and choice of model assumptions can be documented and interrogated, the reasoning behind an LLM's choice of compartmental topology or rate law formulation is neither transparent nor fully reproducible. Further, agents require deep domain‐specific training on QSP literature and best practices, seamless integration with diverse platforms like NONMEM, and regulatory acceptance with transparent decision‐making and human validation at critical checkpoints. Finally, the Virtual Lab framework succeeded in part because nanobody binding can be scored computationally and the scope of the task is narrower. In contrast, QSP for accelerating drug discovery and development is substantially more challenging since the goal is multi‐objective in nature, requiring both cross‐functional and human‐driven contexts at critical junctures of the decision‐making process.

Further advancements in applying agentic AI to QSP modeling could fundamentally shift the field from a boutique, expert‐driven discipline requiring multidisciplinary teams working months to years per model, to a more scalable and accessible practice where individual modelers supported by AI agents can rapidly develop, validate, and deploy models. This would democratize QSP applications, ameliorate resource constraints, and importantly, enable “living” QSP models that can be continuously updated with emerging data and paradigms while maintaining version control and benchmarking against data and earlier models. While significant implementation challenges remain (as discussed above), this evolution positions QSP modelers as strategic scientific orchestrators whose expertise is amplified, analogous to how computational modeling in the 1980s–90s transformed quantitative pharmacology by expanding rather than limiting what individual scientists could achieve.

### 5.2 Making New Discoveries

There is a growing recognition that AI has the potential to broadly transform how scientific discoveries are made [12], with Virtual Lab being an example within drug discovery [11]. To what extent does QSP offer complementary approaches to help make novel discoveries that could aid the pursuit of new drugs?

While high content, multi‐modal data and the associated AI models offer exciting opportunities for uncovering disease mechanisms and identifying potential targets, they can also entice us into implicit assumption that such targets are necessarily causal and that their modulations would reverse the disease, without regarding the compensatory feedback dynamics inherent in human biology and/or pathophysiology. As recently articulated in [13], while single cell omics and virtual cell models offer opportunities to understand dynamics at unprecedented resolutions, they are only local in nature and additional efforts are needed to model system‐wide diseases and therapeutic effects. Agentic AI offers an opportunity to computationally put together cells, tissues, organs, and populations, capture the communications across these multiple levels of organization to recapitulate pathophysiology to a high level of fidelity, and obtain patient‐level insights. If such integrative virtual patients can be constructed to explain a multitude of failed and successful treatments, new QSP‐driven therapeutic discoveries await.

## 6 Outlook

**FIGURE 1:**
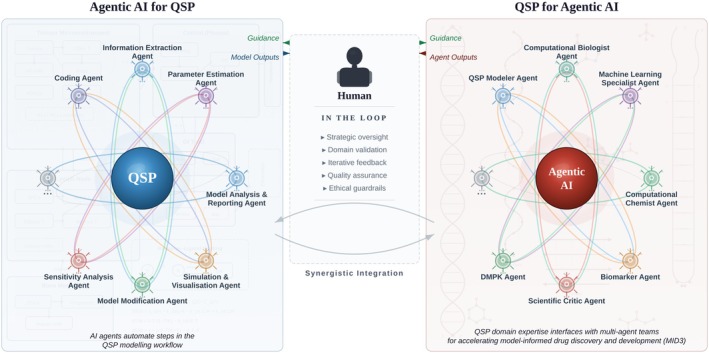
Agentic AI enhances QSP workflows by automating various modeling, simulation, and analysis tasks (left), while QSP perspective and input provide crucial inputs within multi‐agent AI systems to enable model‐informed drug discovery and development (right). It is crucial for humans to remain in the loop and provide oversight across both directions of integration. DMPK, Drug Metabolism and Pharmacokinetics; QSP, Quantitative Systems Pharmacology.

In considering the relationship between QSP and agentic AI, there are two distinct viewpoints that one could take: an *inner view* and an *outer view* [14]; an illustration of these two views is provided in Figure 1. If one adopts an inner view, agentic AI could serve QSP by being considered as part of the compute infrastructure, for instance in performing the roles of information extractor or parameter estimator for QSP modeling. On the other hand, if one adopts an outer view, QSP could serve agentic AI for drug discovery and development by being a hypothesis generator informed by a holistic, systems‐level understanding of view of diseases and pharmacological interventions, or as an integrative predictor of the therapeutic effects of drugs on patients. For instance, while the current Virtual Lab consists of a team of AI agents such as Computational Biologist, Machine Learning Specialist, and so on, one could argue that a QSP Modeler agent is needed to contextualize drug mechanisms within the underlying pathophysiology at a systems level. In this era of rapid change, to fully harness the opportunities offered by agentic AI we should not view QSP as merely a collection of models and algorithms, but more fully as a scientific discipline centered on systems‐level reasoning about drugs and diseases—regardless of whether the hand doing the modeling is that of human or AI (with human‐in‐the‐loop). In this spirit, we hereby invite the community to participate in this and other evolving conversations relating to AI by contributing to CPT:PSP's open call for the upcoming special collection, *Transformative Approaches in Artificial Intelligence for Pharmacometrics and Systems Pharmacology*.

## Funding

The authors have nothing to report.

## Conflicts of Interest

J.L. is employed by A*STAR. R.D. is employed by GSK and owns equity. The contents of this publication reflect R.D.'s own personal opinion and not those of his employer, GSK. The authors declare no other conflicts of interest.
